# Total excision and V-Y plasty technique in the anal area condyloma acuminatum

**DOI:** 10.4103/0970-0358.41115

**Published:** 2008

**Authors:** Arif H. Demirel, Ali U. Ongoren, Ferruh Bingül, Nevzat Gulcelik

**Affiliations:** Department of General Surgery, Ministry of Health Ankara Training and Research Hospital, Ankara, Turkey; 1 Department of Plastic Surgery, Ministry of Health Ankara Training and Research Hospital, Ankara, Turkey

**Keywords:** Condyloma acuminatum, V-Y flap

## Abstract

Condyloma acuminatum is located in the perianal region, anal canal, vagina and the perineum. It is caused by human papillomavirus types 6 and 11. A 18 year-old man was admitted to the clinic because of a perianal mass. On examination of the patient's perianal area and inside the anal canal, a mass was found, which was nearly 8 × 8 cm in size. We could not obtain any information about venereal transmission. The mass was totally excised and the defect was reconstructed with a bilateral V-Y advancement flap. This technique has been used for sacrococcygeal, ischial and other defects but rarely used for condyloma acuminatum. We think that total excision and the use of the V-Y advancement flap technique is safe and has low morbidity in the treatment of condyloma acuminatum.

## INTRODUCTION

Condyloma acuminatum is caused by human papilloma virus (HPV) types 6 and 11 and can be located in the perianal region, anal canal, vulva, vagina and/or the perineum. This is a viral venereally transmitted disease. There is a possibility that this illness can result in cancer and therefore, we must be careful in treatment and aftercare.[1] Total excision and the use of a V-Y advancement flap technique is used in a perianal and anal canal condyloma acuminatum case and a review of the literature is also added.

## CASE HISTORY

**Figure 1:**
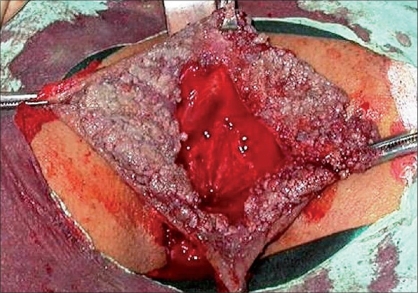
Total excision of mass in anal condyloma acuminatum

**Figure 2:**
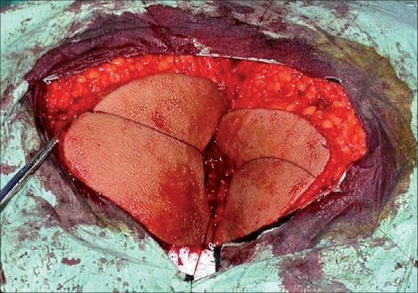
Preparations of bilateral V-Y advancement flap after removal of the lesion

**Figure 3:**
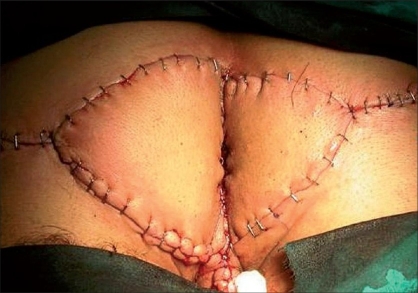
Bilateral V-Y advancement flap in condyloma acuminatum

**Figure 4:**
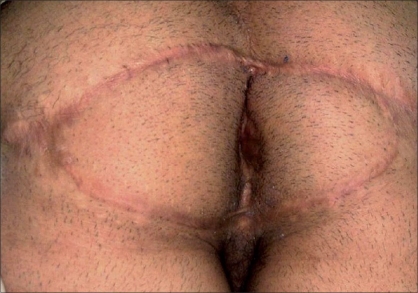
Postoperative appearance 22 months later

A 18-year-old male patient who was complaining about a perianal mass was admitted to our clinic. For two years, the patient had a complaint of itching in the perianal region, painful defecation and constipation. On examination we found a mass measuring 8 × 8 cm in the verrucous form, which reached the dentate line in the anal canal [Figure 1]. No significant medical or familial history was found. We were unable to obtain any information regarding sexual transmission. His blood count and biochemical tests were normal. His serology revealed the following: HBsAg (hepatitis B surface antigen)(+), Anti-HCV (hepatitis C virus)(−), Anti-HIV (human immunodeficiency virus)(−). The mass was separated from the dentate line and totally excised and reconstructed by means of a bilateral V-Y fasciocutaneous advancement flap [Figures 2, 3]. The lateral edges of the flap were sutured with 2/0 polyglactin material, the medial edge of the flap was sutured to the dentate line by a 3/0 polyglactin material. The skin incision was closed with a stapler. There were no postoperative complications and the patient was discharged. The patient was examined 22 months after the operation. Anal continence and the appearance of the flaps and anal canal were normal [Figure 4].

## DISCUSSION

Condyloma acuminatum is a viral illness caused by HPV. The incubation period is between 1-6 months but some authors claim that it can be longer. Condyloma acuminatum is a sexually transmitted disease, however, it can also occur at birth or due to contact with already infected people.[2] Of these cases, 83-90% were homosexual males.[2] Condyloma acuminatum occurs more often in immunosuppressed patients than in immunocompetent patients. Breese reported that HPV infection is common in HIV(+) cases; it is determined that 61% of HIV(+) patients and 17% of HIV(−) patients have anal HPV infection. This is the most common sexually transmitted viral infection in the USA.[1]

Condyloma acuminatum can be located in the perianal region, anal canal, vagina, penis and/or the perineum. In most cases, it is diagnosed by inspection but sometimes it is hard to differentiate between second term syphilis lesions, Condyloma lata and squamous cell carcinomas of the anus.[1]

To treat this illness, topical agents such as podophyllin, podophyllotoxin, bichloroacetic acid, antineoplastic agents can be used and local destructive methods such as cryotherapy, electrocoagulation, laser therapy and surgical excision have also been reported.[1,2] For patients with widespread lesions and also pregnant women, laser treatment can be applied resulting in only 3-14% relapse rates. Immunotherapy is an alternative method in relapsed cases and in cases with widespread lesions.[1]

Excision by scalpel, which has been used for many years for small and multiple lesions has a 20% relapse rate. In some cases, as in our case, where there is a very large lesion which covers the whole anal region, it can be reconstructed by means of skin grafts and flaps after the large total excision.[2] After surgical excision, the relapse rates were between 9-42%. Possible postoperative complications can be bleeding, hematoma and anal stricture. To prevent anal stricture, intact tissue is retained during surgery.[1] In this case, the warts were widespread and total excision was necessary, so we were unable to retain intact tissue. We applied a V-Y advancement flap on the defect. Tuncbilek and colleagues have successfully applied the V-Y advancement flap technique in sacrococcygeal and ischial defects.[3] Liron-Ruiz and colleagues have used this technique for perianal hidradenitis suppurativa treatment.[4] Hassan and Nivatvongs have reported that this technique is useful to reduce perianal defects.[5] Uribe had successful results with this method especially in HIV(+) cases who had giant condyloma acuminatum.[6]

HPV 16 and 18 genomes are responsible for squamous cell carcinoma of the anal area. Although it is not proved that there is a connection between anal area squamous cell carcinoma and condyloma acuminatum, it is found to be prevalent in homosexual males.[2]

As a result, it is important to choose the correct treatment for condyloma acuminatum. We believe that on large lesions in the perianal area and anal canal condyloma acuminatum cases, total excision and the V-Y advancement flap technique can be used safely and with low morbidity.
